# The Soluble Recombinant *Neisseria meningitidis* Adhesin NadA_Δ351–405_ Stimulates Human Monocytes by Binding to Extracellular Hsp90

**DOI:** 10.1371/journal.pone.0025089

**Published:** 2011-09-16

**Authors:** Paola Cecchini, Regina Tavano, Patrizia Polverino de Laureto, Susanna Franzoso, Cristina Mazzon, Paolo Montanari, Emanuele Papini

**Affiliations:** 1 Centro Ricerche Interdipartimentale Biotecnologie Innovative (C.R.I.B.I.), University of Padova, Padova, Italy; 2 Department of Biomedical Science, University of Padova, Padova, Italy; 3 Department of Pharmaceutical Science, University of Padova, Padova, Italy; 4 Novartis Vaccine and Diagnostics srl, Siena, Italy; Ecole Polytechnique Federale de Lausanne, Switzerland

## Abstract

The adhesin NadA favors cell adhesion/invasion by hypervirulent *Neisseria meningitidis* B (MenB). Its recombinant form NadA_Δ351–405,_ devoid of the outer membrane domain, is an immunogenic candidate for an anti-MenB vaccine able to stimulate monocytes, macrophages and dendritic cells. In this study we investigated the molecular mechanism of NadA_Δ351–405_ cellular effects in monocytes. We show that NadA_Δ351–405_ (against which we obtained polyclonal antibodies in rabbits), binds to hsp90, but not to other extracellular homologous heat shock proteins grp94 and hsp70, *in vitro* and on the surface of monocytes, in a temperature dependent way. Pre-incubation of monocytes with the MenB soluble adhesin interfered with the binding of anti-hsp90 and anti-hsp70 antibodies to hsp90 and hsp70 at 37°C, a condition in which specific cell-binding occurs, but not at 0°C, a condition in which specific cell-binding is very diminished. Conversely, pre-incubation of monocytes with anti-hsp90 and anti-hsp70 antibodies did not affected NadA_Δ351–405_ cell binding in any temperature condition, indicating that it associates to another receptor on their plasma membrane and then laterally diffuses to encounter hsp90. Consistently, polymixin B interfered with NadA_Δ351–405_ /hsp90 association, abrogated the decrease of anti-hsp90 antibodies binding to the cell surface due to NadA_Δ351–405_ and inhibited adhesin-induced cytokine/chemokine secretion without affecting monocyte-adhesin binding. Co-stimulation of monocytes with anti-hsp90 antibodies and NadA_Δ351–405_ determined a stronger but polymixin B insensitive cell activation. This indicated that the formation of a recombinant NadA/hsp90/hsp70 complex, although essential for full monocyte stimulation, can be replaced by anti-hsp90 antibody/hsp90 binding. Finally, the activation of monocytes by NadA_Δ351–405_ alone or in the presence of anti-hsp90 antibodies were both inhibited by neutralizing anti-TLR4 antibodies, but not by anti-TLR2 antibodies. We propose that hsp90-dependent recruitment into an hsp90/hsp70/TLR4 transducing signal complex is necessary for the immune-stimulating activity of NadA_Δ351–405_ anti-MenB vaccine candidate.

## Introduction

*Neisseria meningitidis* is a principal cause of sudden death for septic shock and meningitidis [1], [2]. Hypervirulent meningococcal B serotypes, mostly responsible for infections occurring in developed countries, often express the Oligomeric Coiled-coil Adhesin (OCA) NadA (*Neisseria meningitidis* Adhesin A) [3], [4]. NadA predicted structure comprises a COOH terminal domain necessary for anchorage to the outer membrane, an intermediate stalk rich in α helices, with a leucine zipper, and a NH_2_ terminal globular region containing the binding site(s) for a still unknown NadA cellular receptor [5]. The 45 KDa NadA polypeptide assembles in trimers expected to form a super-molecular array on the bacterial surface, similarly to other homologous OCA adhesins [6]. A soluble recombinant deletion mutant of NadA (NadA_Δ351–405_), retaining a native-oligomeric conformation, has been shown to be an effective immunogen in animal models, inducing bactericidal antibodies. Therefore NadA_Δ351–405_ is at present one of the component of a multivalent anti-Men B vaccine under development [7]. Expression of NadA on *E. coli* determines an increase bacterial adhesion to and invasion of conjunctival cells, while its presence on *N. meningitidis* seems to increase epithelial cell invasion prevalently [5].

In addition it has also been observed that human monocytes, macrophages and dendritic cells differentiated *in vitro* from monocytes all express specific binding sites for NadA_Δ351–405_ and are activated by this soluble recombinant adhesin form [8]–[10]. NadA_Δ351–405_ not only is intrinsically immunogenic, but is expected to increase antigen presentation as a specific adjuvant interacting with cell surface receptors. The presence of appropriate co-stimuli, in particular IFNγ or the TLR7 agonist R848, optimizes the cellular effects of soluble NadA. Interestingly, the membrane organization of full length NadA in MenB outer membranes vesicles (OMVs) increases their intrinsic efficacy in stimulating human macrophages antigen presentation machinery but not the induction of shock-related cytokines in circulating monocytes, independently on IFNγ [10], suggesting that pro-immune action of wt NadA might play a role also *in vivo*.

Because of its importance as an anti-MenB vaccine candidate, in this study we engaged in the characterization of the molecular mechanism underlying the activation of myeloid cells by NadA_Δ351–405_. To address this issue, we decided to search for proteins able to bind to the recombinant adhesin present in total lysates from human monocytes, a primary cell type obtainable in great quantity from fresh blood. We show that purified recombinant NadA_Δ351–405_ binds to hsp90 in solution and on the extracellular side of the monocyte plasma membrane were this heat-shock protein was found to be significantly expressed. We proved that hsp90, although not the NadA_Δ351–40_ receptor, is nevertheless engaged in the formation of a post-receptor complex with the purified adhesin, necessary for cell activation and the following induction of cytokine/chemokine production. Experiment with neutralizing antibodies proved that this process also involves TLR4.

## Results

### NadA_Δ351–405_ binds to hsp90

**Figure 1:**
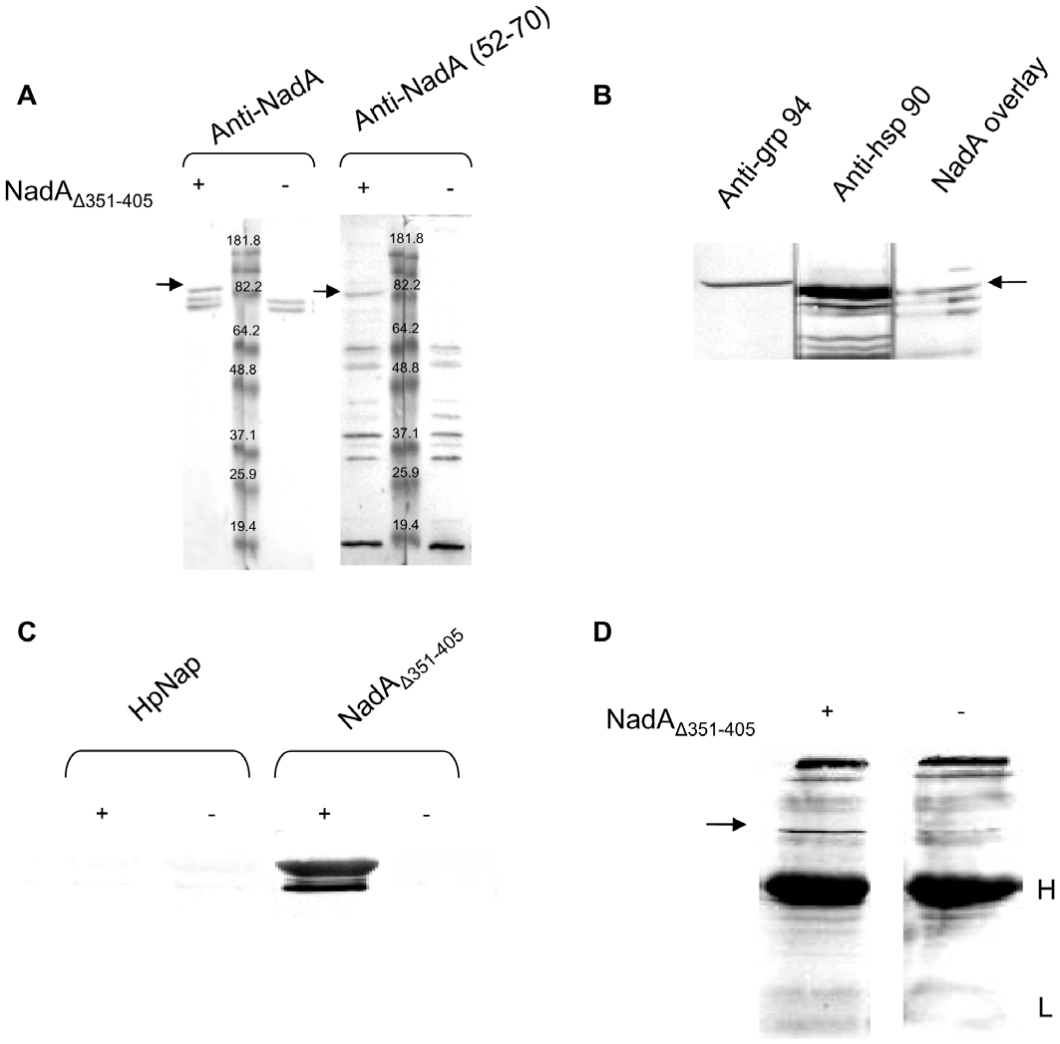
NadA_Δ351–405_ binds to human hsp90 in an overlay-assay. A) Samples of total monocytes extracts obtained with 1% TX-100 were separated by SDS-PAGE and challenged or not as indicated with 1 µM NadA_Δ351–405_ after blotting on nitrocellulose. After extensive washings, the polypeptide bands able to retain NadA were detected with antibodies to the whole adhesin or to NadA 52–70, as indicated. The picture is from a representative experiment out of four and arrows point to the NadA-interacting ∼90 KDa protein. B) Monocytes detergent extracts prepared as in A) were separated by SDS-PAGE, blotted on nitrocellulose and subjected to western blot with anti- grp94 and hsp90 antibodies or to an overlay assay with NadA/anti-NadA antibodies. The mobility of the NadA interacting protein and of hsp90 or grp94 was compared. C) Purified human hsp90 were subjected to overlay assay with (1 µM) or without NadA_Δ351–405_ and HP-NAP as indicated. D) Whole TX-100 extracts from human monocytes were incubated with protein A sepharose-bound anti-NadA IgG in the presence or not of NadA_Δ351–405_. After washings, beads were treated with LSB containing 4%SDS and 10% βme and analyzed by western blot after SDS-PAGE using anti-hsp90 antibodies and alkaline phosphatase-conjugated anti-IgG secondary antibodies. Arrow points to hsp90, H and L indicate antibody heavy and light chains respectively.

Proteins solubilized from human monocytes with TX-100 were separated by SDS-PAGE, blotted on nitrocellulose and incubated or not (controls) with purified NadA_Δ351–405_. NadA-capturing bands were detected using rabbit polyclonal antibodies risen against the whole NadA protein or against the NadA 52–70 sequence and anti-rabbit IgG conjugate to alkaline phosphatase. As shown in Fig 1, A, both anti-NadA antibodies selectively stained a polypeptide of ∼90 KDa, which was absent in control samples. Considering the suggested important role of heat shock protein in microbial factor recognition by immune and epithelial cells, we tested whether this band had the same mobility of hsp90 in SDS-PAGE. Indeed, specific antibodies to hsp90 but not to the homologous grp94 stained a polypeptide having the same mobility of the NadA-interacting protein in a whole monocyte lysates (Fig. 1, B). Hsp 70, detected with a specific polyclonal antibody, migrated with a clearly different molecular weight (not shown). The interaction of NadA with hsp90 was confirmed using purified human hsp90 as capturing protein in an overlay assay (Fig. 1, C). Recombinant Hp-Nap, another bacterial virulence factor known to stimulates monocytes [11], did not bind to purified hsp90 in overlay assays, suggesting that hsp-90 NadA association is protein-specific (Fig. 1, C).

**Figure 2:**
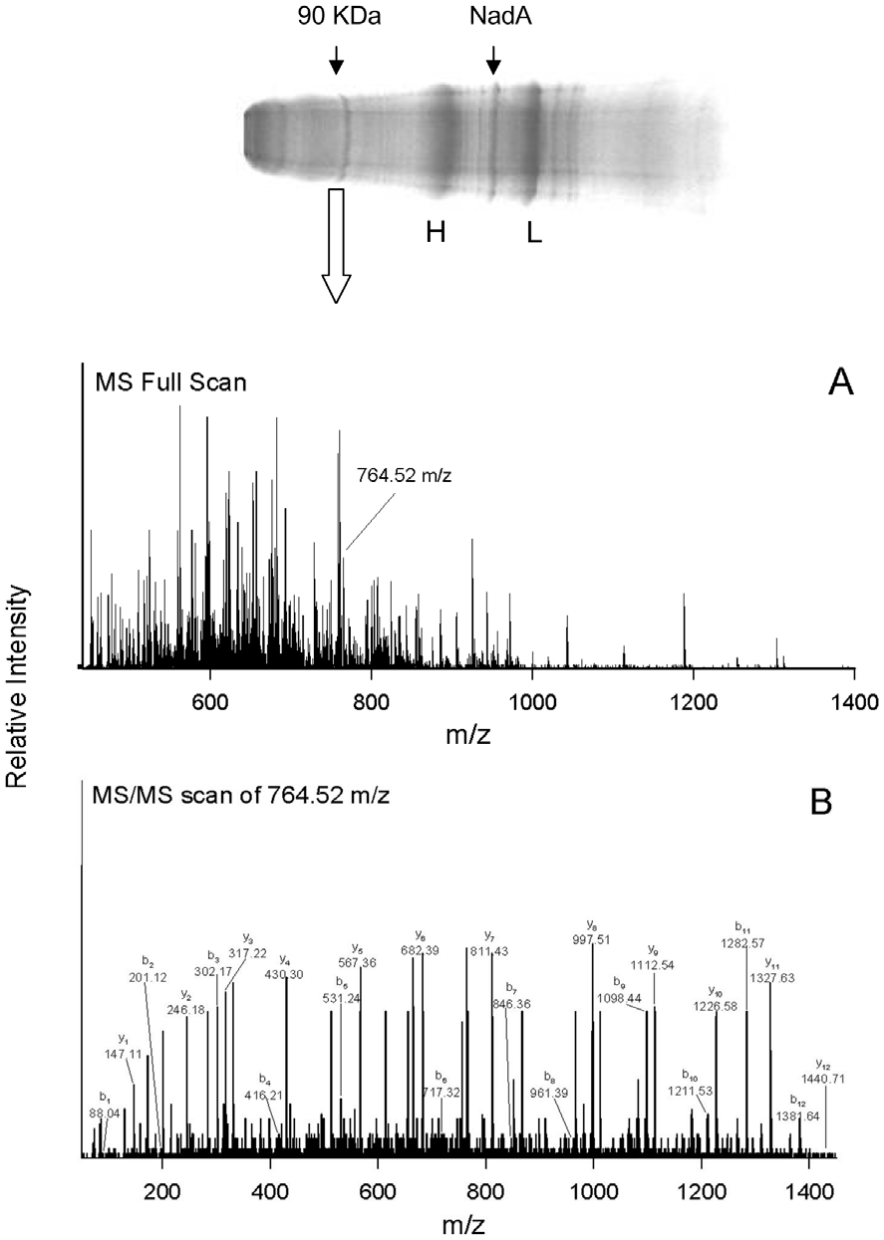
Identification of the 90 KDa protein associated with NadA as hsp90 β by MS/MS. The picture shows a typical Coomassie G250 profile of the proteins co-isolated with NadA/anti-NadA immune complexes. The two arrows point to the 90 KDa protein and to 35 KDa NadA_Δ351–405_, while H and L indicate antibody chains. The 90 KDa was excised from the gel for identification by MS/MS sequencing after trypsinization and identified as the human hsp90 β. The MS full scan (A) and data dependent MS/MS sequencing scans of the high mascot score peptide candidate 764.52 m/z (B) are shown, followed by a table of its Y and B ions (Table 1).

In overlay experiments hsp90 folding is significantly lost. Hence, to determine whether NadA binds also to native hsp90, monocyte total lysates were incubated in non-denaturating conditions with NadA_Δ351–405_ and with anti-NadA antibodies. The immune complexes were isolated from the mixture using proteinA sepharose beads and the co-immune purified proteins were then analyzed by western blot with specific anti-heat shock proteins antibodies. Data shows that native hsp90 co-immune isolated with NadA (Fig. 1, D) while grp94 and hsp70 did not (not shown), in agreement with overlay data. Electron Spray Mass Spectrometry after trypsin digestion of NadA co-immune precipitated 90 KDa band demonstrated definitively that the NadA-binding protein from monocytes was hsp90 and precisely the most abundant β isoforms (Fig. 2, Table 1). No significant presence of peptides form other proteins were detected in correspondence of the eluted 90 KDa band.

**Table 1:** Peptides identified by the MS/MS tandem analysis.

Start-End	m/z Obs	Mr_expt_	Mr_calc_	Delta	Score	Sequence
42–53	638.4603	1274.9060	1274.6354	0.2707	60	R.ELISNASDALDK.I
42–53	638.4755	1274.9364	1274.6354	0.3011	21	R.ELISNASDALDK.I
56–64	520.3580	1038.7014	1038.4869	0.2145	48	R.YESLTDPSK.L
73–82	597.9259	1193.8372	1193.6404	0.1968	53	K.IDIIPNPQER.T
83–95	675.5099	1349.0052	1348.7272	0.2781	52	R.TLTLVDTGIGMTK.A
96–107	621.9464	1241.8782	1241.6979	0.1803	67	K.ADLINNLGTIAK.S
187–196	656.3795	1310.7444	1310.5626	0.1818	67	K.EDQTEYLEER.R
205–219	905.1459	1808.2772	1807.9508	0.3264	75	K.HSQFIGYPITLYLEK.E
205–219	603.7777	1808.3113	1807.9508	0.3605	49	K.HSQFIGYPITLYLEK.E
276–284	576.3849	1150.7552	1150.5506	0.2047	42	K.YIDQEELNK.T
285–291	451.3546	900.6946	900.5181	0.1765	30	K.TKPIWTR.N
**307**–**319**	**764.5203**	**1527.0260**	**1526.7365**	**0.2896**	**92**	**K.SLTNDWEDHLAVK.H**
307–319	510.0168	1527.0286	1526.7365	0.2921	31	K.SLTNDWEDHLAVK.H
320–330	674.9549	1347.8952	1347.6571	0.2382	57	K.HFSVEGQLEFR.A
331–337	415.3512	827.6878	827.5221	0.1657	42	R.ALLFIPR.R
338–347	618.9285	1235.8424	1235.6298	0.2126	32	R.RAPFDLFENK.K
360–378	1187.8018	2373.5890	2373.1384	0.4506	54	R.VFIMDSCDELIPEYLNFIR.G
360–378	792.2254	2373.6544	2373.1384	0.5159	21	R.VFIMDSCDELIPEYLNFIR.G
379–392	757.5038	1512.9930	1512.7783	0.2147	77	R.GVVDSEDLPLNISR.E
539–550	708.9680	1415.9214	1415.6303	0.2911	58	K.EGLELPEDEEEK.K

Peptides identified by the MS/MS analysis and recognized by Mascot. In the columns are indicated, from left to right: the relative sequence position; the experimental m/z value (m/z Obs); the theoretical molecular weight, in Dalton, obtained from the Swiss-Prot and ExPASy databases (Mr_exp_); the relative molecular mass calculated from the matched peptide (Mr_calc_); the difference between the experimental and calculated masses (Delta); the Mascot score for the identified protein (Score) and the sequence of the peptide. The peptide with the highest score is highlighted in bold and used as example of MS/MS scan analysis in Fig. 2. All shown peptides belong to human hsp90 β.

**Figure 3:**
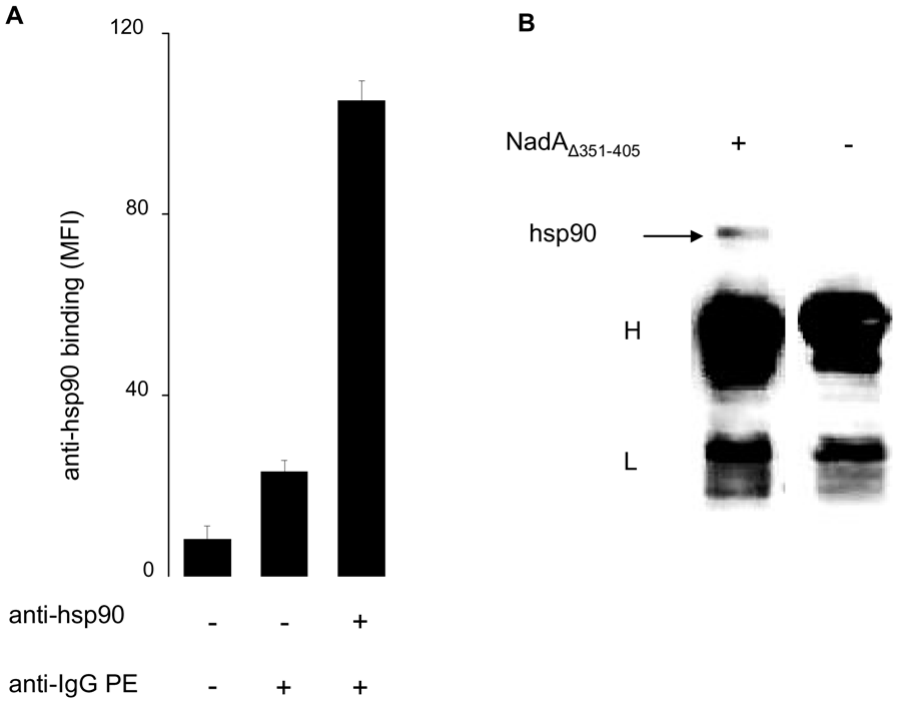
Hsp90 is present on the surface of human monocytes and co-immune isolates with plasma membrane-bound NadA. A) Cells were pre-saturated with human serum to block non-specific antibody binding and then treated with anti-hsp90 and PE-conjugated anti-IgG antibodies for 2 hours at 0°C. Mean fluorescence intensity was quantified by flow-cytofluorimetry. Data are the mean of three experiments run in duplicate and bars represent +/− SE. B) Monocytes were pre-treated or not with NadA_Δ351–405_ (5 µM) for 3 hours at 37°C, washed with PBS at 0°C and further incubated with anti-NadA antibodies (1 µg/ml) at the same temperature. After three hours, cells were washed again, dissolved with 1% TX-100 and protein recovered by protein A-sepharose beads were subjected to western blot with specific anti-antibodies to reveal hsp90 (pointed by an arrow).

Although hsp90 is mostly expressed in the cell cytosol, flow cytofluorimetry analysis demonstrated that monocytes also display a significant fraction of this protein on the plasma membrane (∼5% of total cellular amount), in agreement with its proposed role in the detection of extracellular microbial agonists [12]–[14] (Fig. 3, A). Consistently with this information, co-immune precipitation experiments in which pre-bound NadA_Δ351–405_ was treated with anti-NadA antibodies before detergent monocytes lysis, showed that hsp90 co-isolated with plasma membrane bound MenB adhesin (Fig. 3, B).

### NadA_Δ351–405_ affects the binding of anti-hsp90 antibodies to hsp90 on monocytes

**Figure 4:**
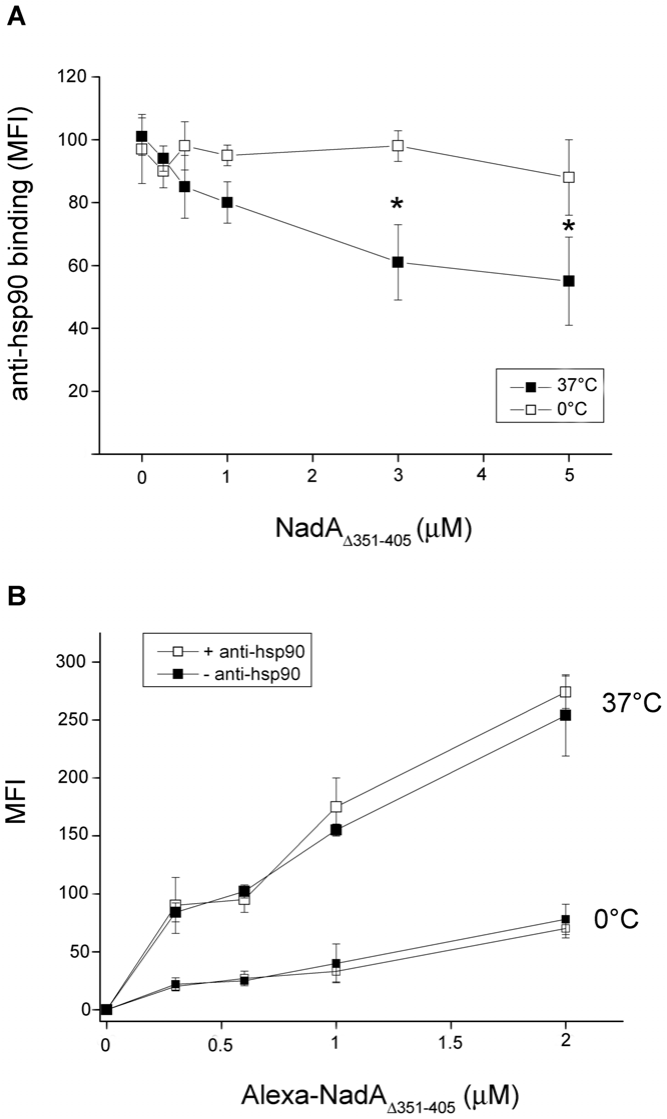
Analysis of the reciprocal interference of NadA_Δ351–405_ and anti-hsp90 antibodies to the monocyte surface at 37° and 0°C. A) Human monocytes were incubated for 1 hours at 0°C in RPMI, 10% FCS, containing NadA_Δ351–405_ at the indicated concentrations. After washing at 0°C cells were further incubated with anti-hsp90 antibody and anti-IgG PE-labeled secondary antibodies. MFI was then analyzed by flow-cytofluorimetry. Data are from an experiment representative of four, and bars represent +/− SE. Asterisk indicates signals significantly different (p<0.05) from control (0°C). B) Monocytes were pre-treated or not with anti-hsp90 antibodies (40 µg/ml) for 1 hour at 0°C, washed as above and further incubated at the indicated temperature with Alexa-labeled NadA_Δ351–405_. Graphs are made with mean values from a representative experiment of four. Bars are +/− SE.

To collect additional information on recombinant NadA-hsp90 interaction on the monocyte plasma membrane we exploited specific antibodies to hsp90. Consistent with NadA-hsp90 close association, we observed that the pre-binding of NadA_Δ351–405_ to cells hampered the subsequent binding of anti-hsp90 antibodies to hsp90 (Fig. 4 A). This effect progressively occurred at concentrations of soluble adhesin in the micromolar range, in agreement with the known affinity of NadA_Δ351–405_-monocyte binding [9]. Moreover, such a competition only occurred when the recombinant adhesin pre-incubation was performed at physiological temperature (37°C), a condition required for efficient specific cell-binding [9] rather than at 0° C. Dose and temperature dependence both suggest that the interference of anti-hsp90 antibodies to hsp90 correlates with specific NadA cell association.

Similar experiments conducted with anti-hsp70 antibodies proved that NadA_Δ351–405_ cell association also interfered with their binding to the plasma membrane, where hsp70 was detected as well (not shown).

### Anti-hsp90 antibodies do not affect NadA_Δ351–405_ binding to monocytes

The interference of NadA_Δ351–405_ with the association of specific antibodies to hsp90 suggests that they both bind to hsp90, in agreement with biochemical evidence. This implies that, if this heat shock protein is the NadA _Δ351–405_ receptor or is close to it, anti-hsp90 antibodies should hamper the binding of the soluble adhesin to cells. For example, anti-hsp90 antibodies were found to inhibit the CD14 mediated transfer of LPS to the hsp90/hsp70/TLR4 signaling multiple complex on human macrophages [13]. However, pre-treatment of monocytes with anti-hsp90 antibodies to both the COOH an the NH_2_ terminal domains, or with a combination of anti-hsp90 and anti-hsp70 antibodies did not affect whatsoever the binding of NadA_Δ351–405_ on human monocytes at both 0°C and 37°C (Fig 4, B). The lack of a reciprocal interference effect of NadA_Δ351–405_ and anti-hsp90 antibodies with respect to their binding to the monocytes surface suggests that the interaction with hsp90 is not required for cell binding of NadA_Δ351–405_.

### Polymixin B inhibits the interaction of NadA_Δ351–405_ with hsp90 but does not affect its association to monocytes

**Figure 5:**
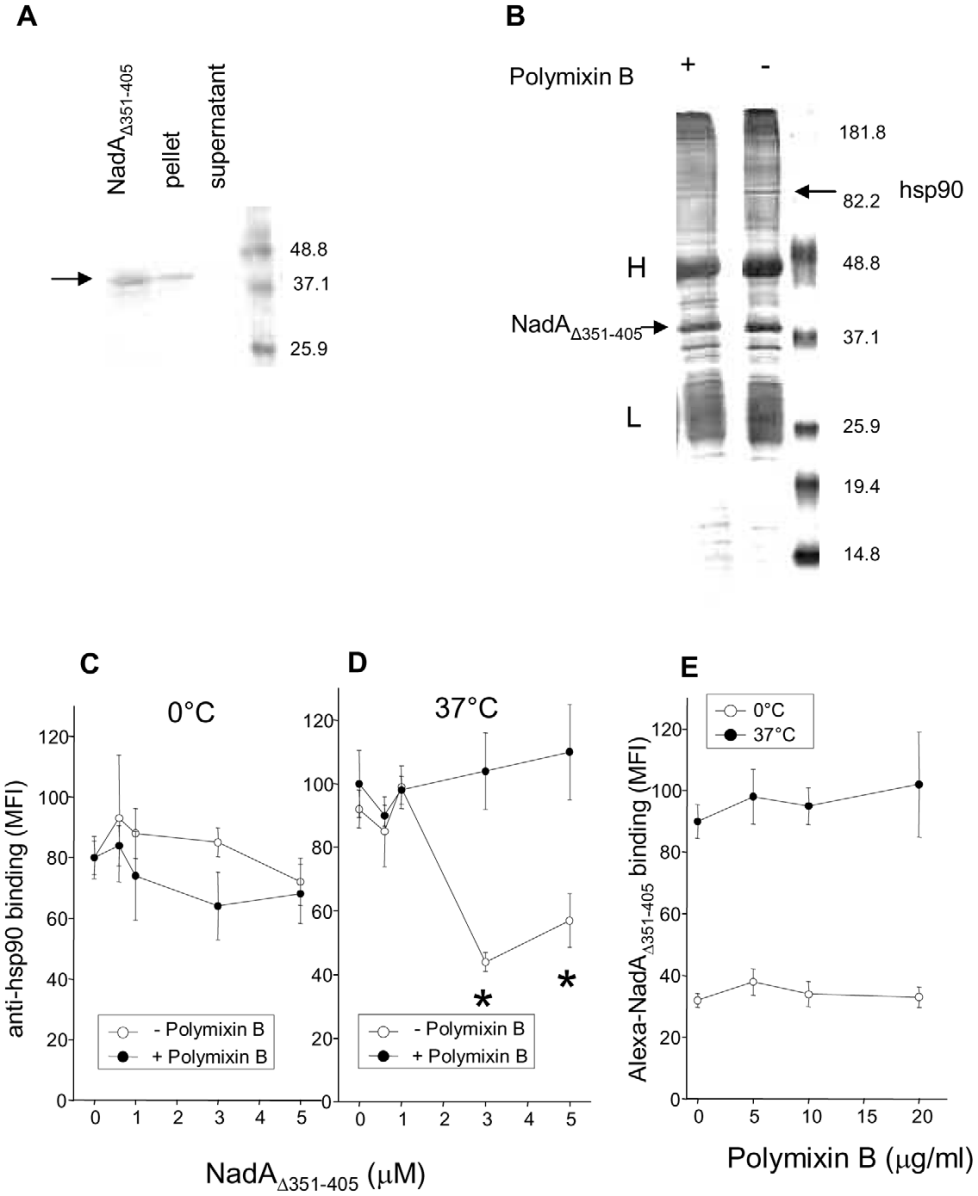
Polymixin B binds to NadA_Δ351–405_, inhibits the formation of the NadA_Δ351–405_/hsp90 complex but does not hamper NadA_Δ351–405_ monocyte binding. A) 0.1 ml of NadA_Δ351–405_ solution in PBS (10 µg/ml) was incubated with polymixin B agarose at room temperature for 2 hours. Beads were recovered by centrifugation, and corresponding supernatant and pellets, washed in PBS, were subjected to SDS-PAGE to reveal NadA presence by Coomassie staining (arrow). A reference lane containing 1 µg NadA_Δ351–405_ was run in parallel. B) Monocyte detergent extracts were subjected to co-immune isolation with proteinA-sepharose bound anti-NadA antibodies and NadA_Δ351–405_ plus or minus 10 µg/ml polymixin B, as indicated. Proteins were separated by SDS-PAGE and silver stained. The arrows points to hsp90 or to NadA; H and L indicate the chains of anti-NadA antibodies. C, D) Monocytes were incubated with the indicated concentrations of NadA_Δ351–405_ at 0° C (C) or 37°C (D) in the presence (black circles) or in the absence (open circles) of 20 µg/ml polymixin B for three hours in culture medium. After washings with PBS, at 0°C, cells were further incubated with anti-hsp90 antibodies and with PE-conjugated secondary antibodies. Cell fluorescence (MFI), quantified by flow-cytofluorimetry, corresponds to the mean of three experiments run in triplicate and bars represent +/− S.E. Asterisk indicates signals significantly different (p<0.05) from control (no adhesin present in the system). E) Monocytes were incubated with 600 nM Alexa NadA_Δ351–405_ at 0°C or 37°C in the presence of increasing concentrations of polymixin B. Cells were washed and treated at 4°C with anti-hsp90 and anti-IgG PE-conjugated antibodies keeping polymixin B in the solutions where necessary. Cell fluorescence (MFI), quantified by flow-cytofluorimetry, corresponds to the mean of three experiments run in triplicate and bars represent +/− S.E.

To gain more information on the possible role of the formation of the NadA_Δ351–405_-hsp90 complex on monocyte, we exploited polymixin B. We in fact proved that this amphipatic cyclic cationic peptide, known to bind to LPS [15], also associates to purified soluble NadA. Our highly purified, LPS free (<0.05 EU/µg), NadA_Δ351–405_ preparation, was incubated with sepharose-linked polymixin B in PBS. After extensive washing the presence of the protein in the matrix was revealed by western blot after SDS-PAGE separation. As shown in Fig 5, A NadA_Δ351–405_ was prevalently found associated to polymixin B sepharose while not in solution. Polymixin B inhibited the co-immune isolation of hsp90 from monocyte detergent extracts, but not the binding of anti-NadA antibody to the adhesin (Fig 5, B). Consistently, the inhibition of anti-hsp90 antibodies binding to hsp90 on the monocyte surface by NadA_Δ351–405_ was abrogated when polymixin B was included in the system. All these evidence indicate that polymixin B binds to NadA_Δ351–405_ and blocks its association with hsp90 (Fig 5, C and D). This notwithstanding, polymixin B did not affect Alexa-NadA _Δ351–405_ binding to monocytes, at either 0°C or 37°C (Fig 5, E). Data collected with polymixin B are consistent with those obtained with anti-hsp90 and anti-hsp70 antibodies and further support that soluble recombinant NadA binds to the monocyte surface thanks to a receptor different from hsp90 and interacts only subsequently with hsp90.

### Polymixin B blocks NadA _Δ351–405_ /dependent cytokine/chemokine secretion by monocytes

**Figure 6:**
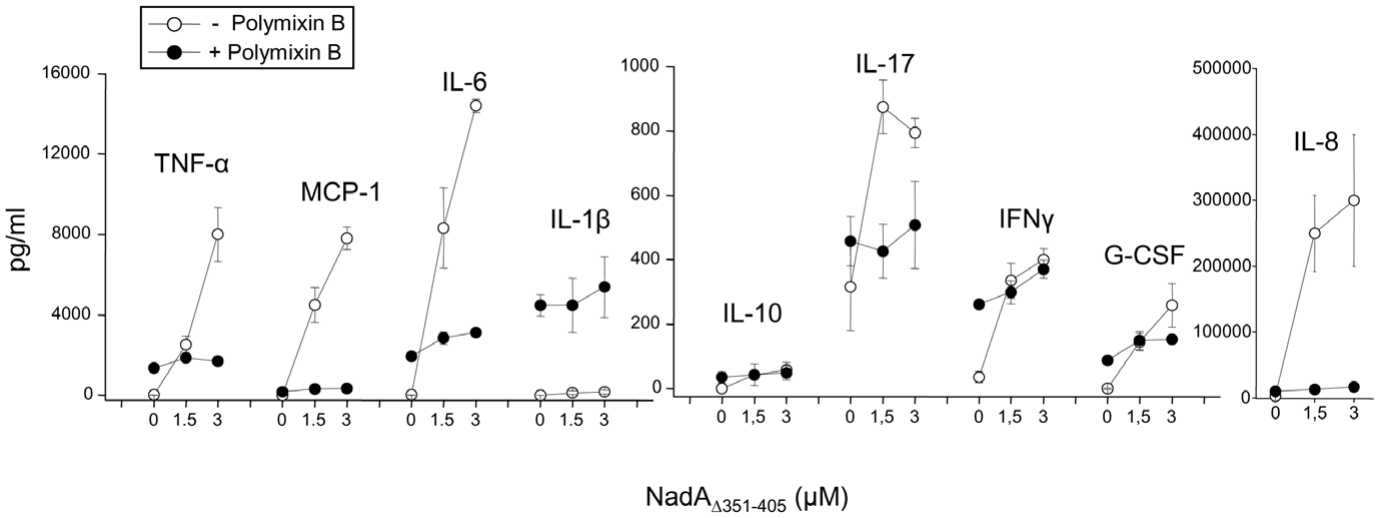
Polymixin B inhibits the induction of cytokine/chemokine by NadA_Δ351–405_ in monocytes. Human monocytes were stimulated for 24 hours with the indicated concentrations of NadA_Δ351–405_ in the absence (open circles) or in the presence of 10 µg/ml polymixin B in DMEM plus 10% FCS. Cytokines/chemokines were subsequently quantified in the cells extracellular medium using a BioPlex assay. Data are from a representative experiment of three, run in duplicate and bars are ranges.

In the previous paragraph we have shown that polymixin B binds to recombinant NadA, interfering with its association to hsp90 while not to monocytes. We therefore decided to use polymixin B as a tool to ascertain the role of hsp90 in monocyte activation induced by NadA_Δ351–405_ [9]. The cytokine/chemokine secretion pattern induced by NadA_Δ351–405_ in monocytes in the presence or not of polymixin B was analyzed in the extracellular medium. Data (Fig. 6) show that polymixin B abrogated NadA-induced secretion of TNFα, MCP-1, IL-6, IL-1B, IL-10, IL-17, G-CSF, IL-8 and MIP-1β. Polymixin B did not inhibit the cytokine inducing activity of HpNap on monocytes a bacterial protein acting through TLR2 [11], a fact that excludes a general impairment of cell activation determined by this substance (not shown).

### Binding of anti-hsp90 antibodies to hsp90 on the surface of monocytes synergizes NadA_Δ351–405_ effects in a polymixin B-insensitive way

**Figure 7:**
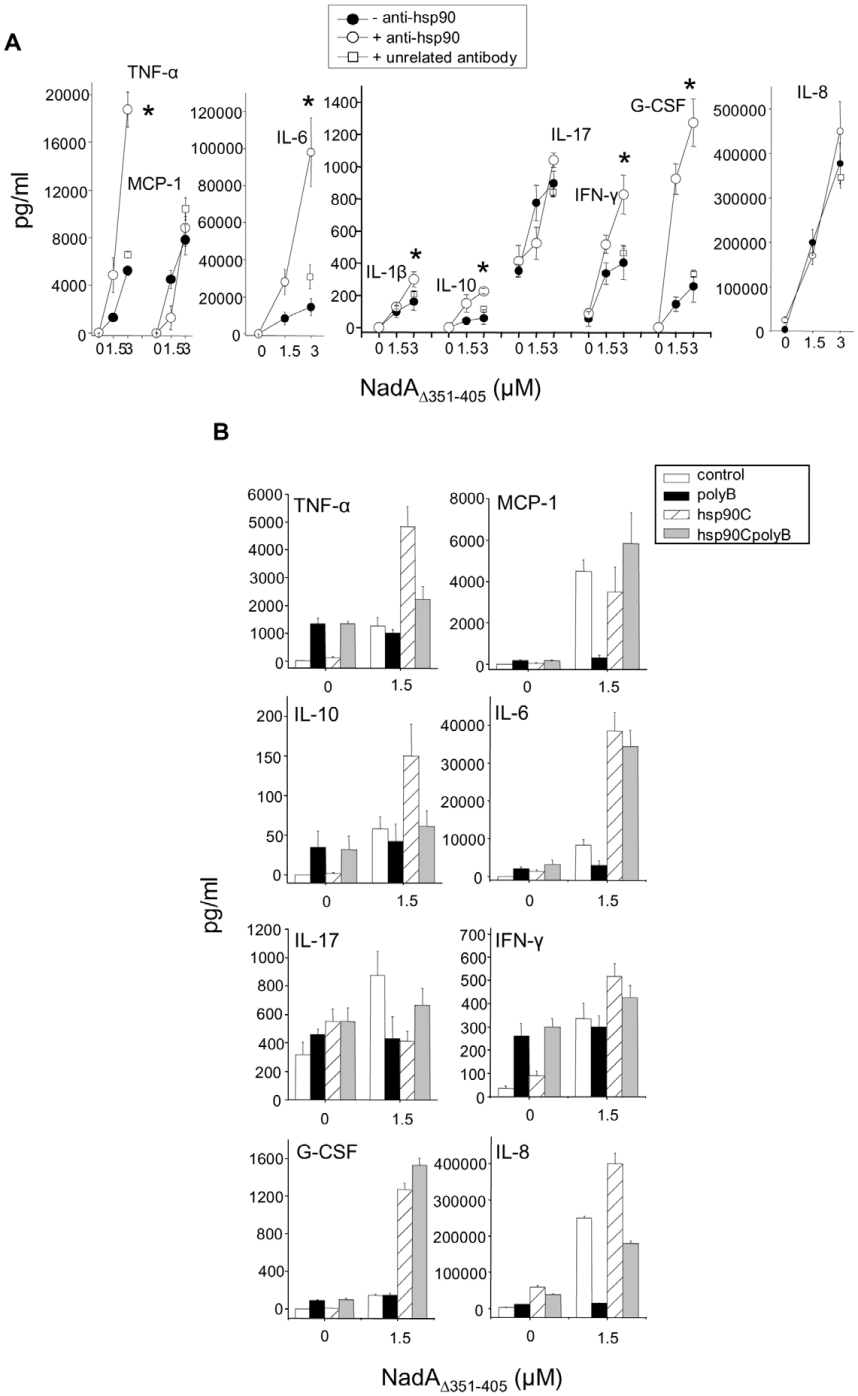
Anti-hsp90 antibodies increase NadA_Δ351–405_ monocyte stimulation in a polymixin B insensitive-way. A) Cells were treated with NadA_Δ351–405_ in the absence (black circles) or in the presence (open circles) of purified rabbit polyclonal antibodies directed to the COOH terminal domain of hsp90. Open squares refers to cells incubated with NadA_Δ351–405_ in the presence of purified rabbit polyclonal antibodies from non immunized animals. The indicated cytokines/chemokines were analyzed in the extracellular medium by BioPlex suspension arrays. Data are the mean from a representative experiment out of four run in triplicate. Bars are +/− SE. Asterisk indicates signals significantly different (p<0.05) from control (with or without an unrelated antibody). B) Monocytes were stimulated or not with NadA_Δ351–405_ (1.5 µM) as indicated, in the presence of polymixin B (black bars), antibodies to the COOH terminal of hsp90 (hatched bats) and in the presence of both polymixin B and anti-hsp90 antibodies (light gray bars). After 1 hour incubation, the indicated cytokines/chemokines were analyzed in the extracellular medium as indicated above. Data are the mean of two experiments run in triplicate. Bars represent +/− SE.

We have previously described how antibodies to hsp90 do not hamper NadA_Δ351–405_ binding to monocytes, consistent with the fact that hsp90 is not the cell receptor for soluble NadA on these cells. Therefore we also measured the effect of anti-hsp90 antibodies on NadA_Δ351–405_ -dependent monocyte activation. Interestingly, co-incubation of cells with NadA_Δ351–405_ and anti-hsp90 antibodies selectively enhanced TNFα, IL-6, IL-10, IFNγ and G-CSF release, and did not alter MCP-1, IL-17 and IL-8 production (Fig. 7, A). Moreover, while monocytes cytokine/chemokine secretion induced by recombinant NadA alone was totally blocked by polymixin B, the effects mediated in the presence of anti-hsp90 antibodies were less or non-sensitive to polymixin B action (Fig. 7, B).

### TLR4 is involved in hsp90/NadA_Δ351–405_ dependent monocyte activation

**Figure 8:**
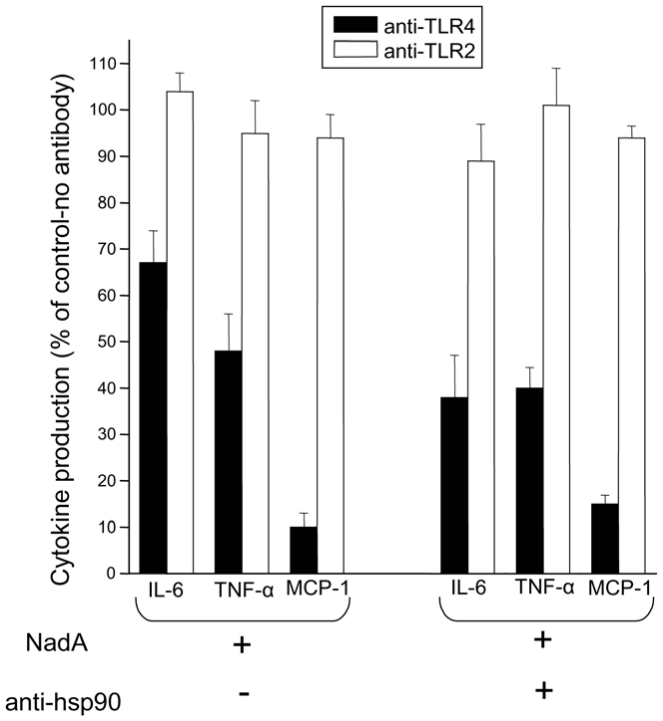
Effect of neutralizing anti-TLR4 and anti-TLR2 antibodies on NadA_Δ351–405_ and NadA_Δ351–405_/anti-hsp90 induced monocyte activation. Cells were incubated with NadA or with NadA and anti-hsp90 antibodies, after pre-treatment with neutralising antibodies to TLR4 or TLR2, as indicated. After 24 hours, the content of the three indicated cytokines was measured in the extracellular medium with a Bio-Plex suspension array. Data are expressed as percentage of the effect induced by adhesin or adhesin plus anti-hsp90 antibodies effects, in the absence of anti-TLR antibodies, and are the mean of three independent experiments run in triplicate +/− SE.

TLR4 has been implicated in the hsp90 mediated activation of inflammatory cells by LPS and taxol [13]. We hence decided to analyze the effect of inhibitory anti-TLR4 and TLR2 monoclonal antibodies on monocyte activation due to NadA_Δ351–405_ alone or in the presence of anti-hsp90 antibodies. We found that only anti-TLR4 antibodies significantly reduced monocytes cytokine release in all condition tested, while anti-TLR2 antibodies were without effects (Fig. 8).

## Discussion

It is known that a recombinant and soluble form of the meningococcal virulence factor NadA (NadA_Δ351–405_) not only is a good immunogen *in vivo*, but is also a immune-stimulant for monocytes and monocytes-derived macrophages and dendritic cells *in vitro*. Such a self-immune enhancing activity may be relevant since NadA has been proposed as a vaccine component in an anti-MenB vaccine formulation. In this work we provide the first insight on the molecular mechanism underlying the effect of such soluble recombinant NadA as a specific agonist. We propose that NadA_Δ351–405_ interaction with extracellular, plasma membrane bound, constitutive hsp90 is a requisite for the following monocyte secretion of cytokines and chemokines. First, we proved that the recombinant NadA binds to hsp90 *in vitro* and on the surface of monocytes, where this heat shock protein, in agreement with other studies (reviewed in [14]) was detected in significant amount. Hsp70 was also measured on monocytes but was not found to bind to NadA_Δ351–405_. Mass spectrometry data confirmed that NadA_Δ351–405_ bound to human hsp90 β isoform. On the contrary Grp94, a membrane heat shock protein belonging to the hsp90 family and interacting with some bacterial virulence factors [16], [17], did not interact with NadA_Δ351–405_. Indeed, when the recombinant NadA was pre-bound to monocytes at physiological temperature (37°C) anti-hsp90 antibodies binding to hsp90 was inhibited with a dose response compatible with its cell-binding affinity. This observation again suggests a contact between the soluble adhesin and hsp90, which inhibits or hinders anti-hsp antibody association to the same protein. Interestingly, in spite of no evidence of NadA_Δ351–405_/hsp70 association, also anti-hsp70 antibody binding to hsp70 on the surface of monocyte was hampered by NadA pre-incubation. This observation is consistent with a close contact between hsp90 and hsp70, and with the proposed existence of a multiple transducing complex comprising these two heat shock proteins and other proteins, involved in activation of inflammatory cells by various microbial agonists. This competition was on the contrary not observed when NadA_Δ351–405_ was pre-bound at 0°C. In a previous study we have shown that specific association of NadA_Δ351–405_ to monocytes was more efficient at 37°C compared to 0°C. Our data seemed therefore to suggest hsp90 is the NadA receptor on monocytes. To test this possibility we studied the effect of anti-hsp90 and anti-hsp70 antibodies pre-incubation on NadA monocyte binding. However, these antibodies did not inhibit NadA_Δ351–405_ binding to monocytes even when used in combination, at both 0° and 37°C. Our data therefore indicate that NadA_Δ351–405_ can associate to hsp90 on monocytes becoming very close to hsp70 as well, presumably thanks to lateral diffusion with the already described hsp90/hsp70 transducing multiple complex [18]–[20], [13], but that this is not relevant for cell binding in monocytes, which is mediated but another still unidentified molecule. Experiments with polymixin B confirmed our view. In fact this molecule was found to bind to NadA_Δ351–405_ and, as a result, to interfere with the formation of the NadA/hsp90 in solution and the surface of monocytes, but was again without any effect on the extent of recombinant NadA-monocyte binding at 0°C and 37°C.

In the second part of our manuscript we focused on the possibility that the formation of the NadA_Δ351–405_ -hsp90 complex, although not necessary for cell binding, is nevertheless necessary for cell activation. In fact NadA is well known to stimulate monocytes [9]. To address this issue we used polymixin B and anti-hsp90 antibodies.

We found that polymixin B efficiently blocks cytokine/chemokine secretion induced by NadA_Δ351–405_, indicating that binding to monocytes without the subsequent engagement of hsp90 is not sufficient to stimulate cells.

Surprisingly, we found that co-incubation of NadA_Δ351–405_ with anti-hsp90 antibodies synergized the effects of the men B adhesin in a selective way increasing the secretion of most, although not all, cytokines/chemokines. In addition, cell activation due to the presence of both agonists was much less or not at all sensitive to the presence of polymixin B. We conclude that binding of anti-hsp90 antibodies to hsp90 can vicariate the need for NadA_Δ351–405_ interaction with the same protein. Since hsp90 was not strongly stimulatory *per se*, at least at these concentrations, this implies that NadA_Δ351–405_ cell association to sites other to hsp90 insensitive to polymixin B, is necessary, although not sufficient, for monocyte stimulation. It remains that, in physiological conditions hsp90 involvement appears necessary for the immune effects induced by recombinant NadA. Finally, using specific anti-TLRs neutralizing antibodies, we showed that cytokine/chemokine induction by NadA_Δ351–405_ requires the transmembrane signaling operated by TLR4, but not by TLR2.

One aspect of our data deserves a special consideration: the analogy between NadA_Δ351–405_ and LPS effects. Both agonists induce cytokines/chemokines by monocytes in a polymixin B sensitive and TLR4 dependent way. This may indicate that a small amount of LPS below the detection limit of our assay is indeed complexed with NadA_Δ351–405_ and is responsible for its interaction with polymixin B and for its biological effects on monocytes. However, we have already proved in our previous work that NadA_Δ351–405_ effect on monocytes is temperature sensitive and that its quantitative elimination from the solution by immune precipitation with specific antibodies abolishes such activity [9]. Moreover, in this manuscript we showed that while NadA_Δ351–405_-induced cytokine production was polymixin B sensitive the enhanced action of NadA plus anti-hsp90 antibodies was not. Finally, biochemical evidence show that all NadA_Δ351–405_ trimers bind to a polymixin B matrix in solution so implying that LPS, to account for such binding, should be complexed with the protein in an equimolar ratio and therefore present in detectable amount in our preparations. For all these arguments we believe that indirect evidence make the possibility that LPS is the real actor of pro-inflammatory effect displayed by NadA_Δ351–405_ preparation very unlikely.

**Figure 9:**
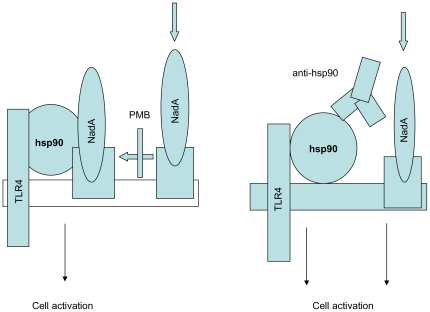
Model of the role of NadA_Δ351–405_/hsp90 interaction in monocyte activation (discussed in the text).

Fig. 9 details the model of monocyte activation by the recombinant NadA we propose. According to our view, the men B adhesin soluble form binds to a till unknown plasma membrane receptor and then, presumably by lateral diffusion, it encounters hsp90 and is recruited into a complex also comprising hsp70 and TLR4. Such association, necessary for full cell stimulation is inhibited by polymixin B, which interferes with NadA_Δ351–405_/hsp90 interaction but not with NadA_Δ351–405_ cell binding. Co-stimulation with anti-hsp90 antibodies not only abolishes polymixin B sensitivity of NadA_Δ351–405_ effects, i.e. the need for NadA_Δ351–405_/hsp90 association, but, paradoxically, optimizes the production of cytokines/chemokines. Our data are reminiscent of the proposed model of LPS-LTA activation of inflammatory cells proposed by [13]. These authors demonstrated that a complex formed by hsp90/hsp70/TLR4/TLR2, and possibly other components, accepts endotoxin after its binding to the plasma membrane LPS co-receptor CD14. In that case however, saturation with anti-hsp90/hsp70 antibody impeded LPS binding and stimulation to the transducing system. On the contrary, we here propose that engagement of hsp90 with specific antibodies mimics the interaction of the adhesin itself with the same protein, which occurs in physiological conditions. In the case of NadA_Δ351–405_, hsp90/TLR4 involvement, although not sufficient to stimulate cells per se, generates a synergy with the effects mediated by the NadA/NadA receptor complex able to stimulate monocytes. In conclusion, our data show that surface-bound hsp90 is a central player in the immune-modulatory effects mediated by the soluble form of NadA selected as a vaccine candidate against *N. meningitidis B*.

Our data indicates that targeting of surface hsp90 may be important to modulate or enhance the efficacy of vaccines or to induce an adjuvant effect, consistent with a general role of hsp90 as an innate immune regulator. On the other hand, since NadA expressing model bacteria or OMVs also show enhanced pro-immune/pro-inflammatory activity compared to negative strains or to NadA lacking OMVs [9], [10] our data allows the hypothesis that the interaction with hsp90 can take place also in the case of native-full-length NadA and may be relevant in the cell interaction of NadA + *N. meningitidis* cells during infection.

## Materials and Methods

### Ethics Statement

Peripheral blood mononuclear cells utilized in this study derived from buffy coats obtained from healthy blood donors, as anonymously provided by the Transfusional Center of the Hospital of Padova. Written informed consent for the use of buffy coats for research purposes was obtained from blood donors by Transfusional Center. Data related to human samples were all analyzed anonymously. Human leukocytes were obtained not consequently to experimentation on human beings but as a consequence of voluntary and informed blood donation for transfusions: no approval of Ethics Committee is needed in such cases in our institution.

This study was conducted according to European Union guidelines for the handling of laboratory animals (86/609 CEE normative, Italian legislative act 116/92). Immunization protocol for antibody production in rabbits (purchased from Harlan Laboratories company) were approved by the Veterinary Service of the University of Padova (project name: Immunoglobulin generation against specific regions of prokaryotic and/or eukaryotic proteins in rabbits – notified to the Italian Health Ministry on 13th October 2006- responsible prof. Emanuele Papini). The Italian legislation for experiments of antibodies production like the ones performed in rabbits in our work foresees the rule of silence/assent with the Minister (Decreto Legislativo 116/92). Moreover at the time 2006–2009 the ethical committee for animal experimentation, which can be activated on a voluntary base by Italian universities according to the legislation, was not present yet in our university. For this reason we have no protocol number ID or any formal approval.

### NadA and labeling of NadA_Δ351–405_ to Alexa

Soluble recombinant NadA was designed as previously described and purified to clinical trial standard [3]. In brief, the DNA sequence of *nadA* allele 3, cloned from the hypervirulent *N. meningitidis* B strain 2996, encoding the deletion mutant NadA_Δ351–405_, with no outer membrane anchor, was cloned into a pET21b vector (Novagen). The protein secreted in the extracellular medium of the transformed *Escherichia coli* BL21(DE3)- NadA_Δ351–405_ strain was purified by Q Sepharose XL, Phenyl Sepharose 6 Fast Flow (Pharmacia) and Hydroxyl apatite ceramic column (HA Macro-Prep, BioRad) chromatographies. Preparations of purified NadA_Δ351–405_ showed a single 35 KDa band after SDS-PAGE and silver staining, consistent with the predicted molecular weight., and is a homotrimer, as assessed by light scattering analysis [5]. Reverse-phase separation of NadA_Δ351–405_ preparation in denaturating conditions showed a single peak homogeneously corresponding to the 1–315 sequence of mature NadA protein, as determined by MALDI-TOFF mass spectrometry analysis. LPS contamination (tested by *Limulus* test kit from Sigma-Aldrich) was <0.005 EU/µg of protein. Bacterial DNA contamination (determined by an enzymatic assay) was 0.6–0.7 pg/µg of protein. No *E. coli* Ags were detected by Western immunoblot analysis with a rabbit polyclonal Ab risen against whole *E. coli* cells (DakoCytomation). Aliquots of protein solution (2 mg/ml in PBS, pH 7.4) were frozen in liquid nitrogen and stored at –80°C. NadA_Δ351–405_ concentration was expressed based on the monomer molecular weight.

NadA_Δ351–405_ was conjugated to the fluorescent probe Alexa 488 using a *N*-hydroxysuccinimidyl derivative (Molecular Probes) according to the manufacturer's instructions. Alexa- NadA_Δ351–405_ was separated from left reagents by size exclusion chromatography using Sephadex G25 (Sigma-Aldrich) columns pre-equilibrated and eluted with PBS at room temperature.

### Antibodies

Rabbit anti-hsp90(H114), against peptide 610–723 of C-terminal human hsp90, goat anti-hsp90(N17), against N-terminal region of human hsp90, and goat anti-hsp70(K20), against a C-terminal peptide of human hsp70, antibodies (Abs) were obtained from Santa Cruz Biotechnology. Anti-human TLR4 and anti-human TLR2 monoclonal Abs were obtained from eBioscence (USA). Secondary Abs (goat anti-rabbit AP- or PE conjugated, mouse anti-goat PA- or FITC conjugated) were from Molecolar Probes.

### Generation and purification of rabbit polyclonal anti-NadA_Δ351–405_ and anti-NadA peptides Abs

Polyclonal antiserum against NadA_Δ351–405_ or 52–70 NadA peptide were generated by immunizing male white rabbits (Harlan Laboratories) with 50 µg of whole protein or 2 mg purified peptide conjugated with 2 mg of keyhole limpet hemocyanin (KLH, Pierce): soluble NadA or a fourth of peptide-KLH complex were homogenized with complete Freund's adjuvant (Sigma) and used to immunize rabbits by cutaneous injection. Three boosters (50 µg of NadA and an aliquot of peptide, homogenized with incomplete Freund's adjuvant- Sigma) were given at days 18, 34, and 52 by intramuscular injection. Antiserum was harvested on day 68 and stored at −20°C.

NadA_Δ351–405_ Abs were obtained using Protein A-Sepharose beads (Pharmacia Biotech); 52–70 NadA peptides Abs were purified using an affinity chromatography, prepared with 52–70 NadA peptide covalent bound to sulfolink coupling gel (Pierce).

### Ligand overlay assay

Cells (10^6^) were solubilized in 100 µl lysis buffer (1% v/v Triton X-100 in PBS) containing a mix of protease inhibitors (Roche diagnostic) for 30 min at 4°C. Lysates were then centrifuged for 5 min at 15.000g to separate cell debris and nuclei. Samples were mixed with Laemmli sample buffer (LSB) and boiled for 5 min at 95°C. Proteins were fractionated on 12% SDS-polyacrylamide gel, blotted onto nitrocellulose membrane and blocked in TBS (50mM Tris-HCl, pH7.5, 100 mM NaCl) –0.1% Tween 20 containing 1% BSA (blocking buffer) at room temperature for 2h. Blots were then incubated in blocking buffer containing or not 1 µM NadA_Δ351–405_ for 8h at 4°C under gently shaking. After 3 washings in TBS–0.1% Tween 20, blots were incubated with anti-NadA_Δ351–405_, anti-NadA peptides, anti-hsp90 or anti-grp94 Abs, and then with secondary PA-conjugated Abs. Bound proteins were detected with NBT-BCIP solutions (Sigma).

### Co-immunoprecipitation

10×10^6^ cells were solubilized in 1 ml lysis buffer containing protease inhibitors at 4°C for 30 min and then lysates were centrifuged for 5 min at 15.000*g*. Supernatants were then incubated with Protein A-Sepharose, previously bound to anti-NadA_Δ351–405_ antibodies and NadA_Δ351–405_; co-immunoprecipitation was performed for 2 hours at 4°C. In some experiments, co-immunoprecipitation was performed in the presence of 10 µg/ml polymixin B. Alternatively, monocytes were incubated for 3 h at 37°C with 5 µM NadA_Δ351–405_ in RPMI-1640, 10% FCS, 50 µg/ml gentamicin and then with anti-NadA_Δ351–405_ Abs (1 µg/ml) for 3 hours. Cells were scraped and lysed and incubated with Protein A-sepharose beads. After 2 hours incubation, beads were washed, resuspended in LSB and analyzed by western blotting or protein staining.

### In-Gel Digestion, MS/MS protein identification and database search

Excised from the gel, spot was washed with 50% *v*/*v* acetonitrile (ACN) in 0.1 M NH_4_HCO_3_, and vacuum-dried. The gel fragments were reduced for 45 min at 55°C in 10 mM DTT in 0.1 M NH_4_HCO_3_. After cooling, the DTT solution was immediately replaced with 55 mM iodoacetamide in 0.1 M NH_4_HCO_3_. After washing with 50% ACN in 0.1 M NH_4_HCO_3_, the dried gel pieces were swollen in a minimum volume of 10 µl digestion buffer containing 25 mM NH_4_HCO_3_ and 12.5 ng/l trypsin (Promega) and incubated overnight at 37°C. Tryptic-digested peptides were extracted according to the protocol described by Bozzo et al. [21].

Protein digestion was performed according to Shevchenko et al. [22] with minor modifications. The dried tryptic digest samples were reconstituted in 10 µl of 0.5% TFA in water and were purified with a ZipTip_C18_ (Millipore). For electrospray MS analysis, the peptides were eluted in 50% acetonitrile containing 0.2% formic acid. Data were collected on a Micromass Q-Tof Micro mass spectrometer (Manchester, UK) (capillary voltage: 3000–3200 V; cone voltage: 45 V; scan time: 1 s; interscan: 0.1 s). Spectra were analyzed using Micromass MassLynx V4.1 software. The MASCOT program (Matrix Science, www.matrix-science.com) was used to search for all MS/MS spectra against the Swiss-Prot database. The following parameters were used in the MASCOT search: trypsin specificity; maximum number of missed cleavages: 1; fixed modification: carbamidomethyl (Cys); variable modifications: oxidation (Met); peptide mass tolerance: ±1.0 Da; fragment mass tolerance: ±0.5 Da; protein mass: unrestricted; mass values: monoisotopic.

### Cell culture

Monocytes were obtained from buffy coats from healthy donors. Leukocyte-enriched plasma was centrifuged over Ficoll-Hypaque (Amersham Biosciences) and Percoll (Amersham Pharmacia Biotech). Mononuclear cells were washed, resuspended in RPMI medium (Gibco) supplemented with 2% fetal calf serum, 50 µg/ml gentamicin, and plated onto a 24-well plate (Falcon). After 1 h-incubation at 37°C in a 5% CO_2_ atmosphere, non adherent cells were removed. The purity of preparations (percentage of CD14-positive cells) and cell viability (using the Trypan blue exclusion test) were higher than 98%. All cells were kept at 37°C in a humidified atmosphere containing 5% (v/v) CO_2_ unless otherwise specified.

### Binding of NadA_Δ351–405_ to polymyxin B

3×10^5^ cells were solubilized in 1 ml lysis buffer containing protease inhibitors at 4°C for 30 min; lysates were then centrifuged for 5 min at 15000*g*. 200 µl of polymyxin B-agarose resin (Sigma-Aldrich) for each sample were incubated 1 h at room temperature under gently shacking with NadA_Δ351–405_ or with lysates. After 2 washing in PBS the supernatant and the resin were mixed with LSB, 5% b-mercaptoethanol and boiled for 5 min at 95°C. Proteins were fractionated on 12% SDS-polyacrylamide gel and Coomassie stained.

### Flow cytometry analysis of hsp90 expression

To quantify hsp90 expression on the surface of monocytes, 5×10^5^ cells were treated for 30 min at 37°C with human serum, to block the Fc receptor expressed on the surface of monocytes, and then for 1h at 4°C with 10 µg/ml of anti-hsp90 Ab. After washing, cells were incubated for 1h at 4°C with 10 µg/ml of PE-conjugated Ab. Total amount of hsp90 was measured in the same way after cells permeabilization, performed by treating cells 15 min in 0.25% paraformaldehyde at room temperature and 1h at 4°C in methanol.

In some experiments, monocytes were pre-incubated for 3h at 4 or 37°C with different concentrations of NadA_Δ351–405_ (from 0.6 to 5 µM), in the presence or in the absence of 10 µg/ml polymyxin B, in RPMI-1640, 10% FCS, 50 µg/ml gentamicin. After three washings in PBS, monocytes were incubated 1h at 4°C with 10 µg/ml anti-hsp90(H114), anti-hsp90(N17) and anti-hsp70(K20) Abs in PBS. Cells were subsequently washed and then treated 1h at 4°C with the appropriated secondary Abs.

After the specific treatments, cells were washed, re-suspended in PBS/1% FCS and analyzed on a FACS Calibur™ flow cytometer (BD Bioscience), adding propidium iodide to eliminate dead cells. Data were collected on 10000–20000 events.

### Bioplex multiplex cytokine assay

Human adherent monocytes were incubated for 24 h in RPMI plus 10% FCS, 50 mg/ml gentamicin, with or without different concentration of NadA_Δ351–405_ (from 0.6 to 3 µM), or/and 10 µg/ml anti-hsp90 (N and C terminal) Abs, or/and 10 µg/ml anti-hsp70 Abs, or/and 10 µg/ml anti-TLR2 or anti-TLR4 Abs, or/and 10 µg/ml polymixin B, and then culture supernatants were collected for performing the assay. A Procarta 22- or 3- cytokines assay kit (Panomix) was used and the assay was performed according to the manufacturer's instructions. Calibration curves from recombinant cytokine standards were prepared with four-fold dilution steps in RPMI-1640 medium containing 10% FBS, 50 µg/ml gentamicin. All assays were carried out in 96-well sterile, prewetted filter plates at room temperature and protected from light. A mixture containing 5000 microspheres per cytokine was incubated together with standards or samples in a final volume of 100 µl for 30 min under continuous shaking (300 rpm). After three washes by vacuum filtration with the washing buffer, a cocktail of biotinylated antibodies diluted in the detection antibody diluent was added. After 30 min incubation and washing, Streptavidin-PE diluted in the assay buffer was added. At the end of 30 min incubation, under continuous shaking and after washing, the fluorescence intensity of the beads was measured in a final volume of 120 µl of the assay buffer. Data analysis was done with Bio-Plex Manager software using a five-parametric curve-fitting. The detection limit of the assay for all antigens was 1 pg/ml.

## References

[1] Mercier JC, Beaufils F, Hartmann JF, Azema D (1988). Hemodynamic patterns of meningococcal shock in children.. Crit Care Med.

[2] van Deuren M, Brandtzaeg Pvan der Meer JW (2000). Update on meningococcal disease with emphasis on pathogenesis and clinical management.. Clin Microbiol Rev.

[3] Comanducci M, Bambini S, Brunelli B, Adu-Bobie Y, Aricò B (2002). NadA, a novel vaccine candidate of Neisseria meningitidis.. J Exp Med.

[4] Comanducci M, Bambini S, Caugant DA, Mora M, Brunelli B (2004). NadA diversity and carriage in Neisseria meningitidis.. Infect Immun.

[5] Capecchi B, Adu-Bobie J, Di Marcello F, Ciucchi L, Masignani V (2005). Neisseria meningitidis NadA is a new invasin which promotes bacterial adhesion to and penetration into human epithelial cells.. Mol Microbiol.

[6] Hoiczyk E, Roggenkamp A, Reichenbecher M, Lupas AHeesemann J (2000). Structure and sequence analysis of Yersinia YadA and Moraxella UspAs reveal a novel class of adhesins.. EMBO J.

[7] Giuliani MM, Adu-Bobie J, Comanducci M, Aricò B, Savino S (2006). A universal vaccine for serogroup B meningococcus.. Proc Natl Acad Sci U S A.

[8] Mazzon C, Baldani-Guerra B, Cecchini P, Kasic T, Viola A (2007). IFN-gamma and R-848 dependent activation of human monocyte-derived dendritic cells by Neisseria meningitidis adhesin A.. J Immunol.

[9] Franzoso S, Mazzon C, Sztukowska M, Cecchini P, Kasic T (2008). Human monocytes/macrophages are a target of Neisseria meningitidis Adhesin A (NadA).. J Leukoc Biol.

[10] Tavano R, Franzoso S, Cecchini P, Cartocci E, Oriente F (2009). The membrane expression of Neisseria meningitidis adhesin A (NadA) increases the proimmune effects of MenB OMVs on human macrophages, compared with NadA- OMVs, without further stimulating their proinflammatory activity on circulating monocytes.. J Leukoc Biol.

[11] Amedei A, Cappon A, Codolo G, Cabrelle A, Polenghi A (2006). The neutrophil-activating protein of Helicobacter pylori promotes Th1 immune responses.. J Clin Invest.

[12] Byrd CA, Bornmann W, Erdjument-Bromage H, Tempst P, Pavletich N (1999). Heat shock protein 90 mediates macrophage activation by Taxol and bacterial lipopolysaccharide.. Proc Natl Acad Sci U S A.

[13] Triantafilou K, Triantafilou M, Ladha S, Mackie A, Dedrick RL (2001). Fluorescence recovery after photobleaching reveals that LPS rapidly transfers from CD14 to hsp70 and hsp90 on the cell membrane.. J Cell Sci.

[14] Schmitt E, Gehrmann M, Brunet M, Multhoff GGarrido C (2007). Intracellular and extracellular functions of heat shock proteins: repercussions in cancer therapy.. J Leukoc Biol.

[15] Morrison DC, Jacobs DM (1976). Binding of polymyxin B to the lipid A portion of bacterial lipopolysaccharides.. Immunochemistry.

[16] Prasadarao NV, Srivastava PK, Rudrabhatla RS, Kim KS, Huang SH (2003). Cloning and expression of the Escherichia coli K1 outer membrane protein A receptor, a gp96 homologue.. Infect Immun.

[17] Cabanes D, Sousa S, Cebria A, Lecuit M, Garcia-del Portillo F (2005). Gp96 is a receptor for a novel Listeria monocytogenes virulence factor, Vip, a surface protein.. EMBO J.

[18] Reyes-Del Valle J, Chavez-Salinas S, Medina FD, el Angel RM (2005). Heat shock protein 90 and heat shock protein 70 are components of dengue virus receptor complex in human cells.. J Virol.

[19] Guerrero CA, Bouyssounade D, Zarate S, Isa O, Lopez T (2002). Heat shock cognate protein 70 is involved in rotavirus cell entry.. J Virol.

[20] Jin S, Song YC, Emili A, Sherman PM, Chan VL (2003). JlpA of Campylobacter jejuni interacts with surface-exposed heat shock protein 90alpha and triggers signalling pathways leading to the activation of NF-kappaB and p38 MAP kinase in epithelial cells.. Cell Microbiol.

[21] Bozzo C, Spolaore B, Toniolo L, Stevens L, Bastide B (2005). Nerve influence on myosin light chain phosphorylation in slow and fast skeletal muscles.. FEBS J.

[22] Shevchenko A, Tomas H, Havlis J, Olsen JV, Mann M (2006). In-gel digestion for mass spectrometric characterization of proteins and proteomes.. Nat Protoc.

